# Generation of four postmortem dura-derived iPS cell lines from four control individuals with genotypic and brain-region-specific transcriptomic data available through the BrainSEQ consortium

**DOI:** 10.1016/j.scr.2020.101806

**Published:** 2020-04-20

**Authors:** Tomoyo Sawada, Kynon J.M. Benjamin, Anna C. Brandtjen, Ethan Tietze, Samuel J. Allen, Apuã C.M. Paquola, Joel E. Kleinman, Thomas M. Hyde, Jennifer A. Erwin

**Affiliations:** a Lieber Institute for Brain Development, Baltimore, Maryland, USA; b Department of Neurology, Johns Hopkins School of Medicine, Baltimore, Maryland, USA; c Department of Psychiatry & Behavioral Sciences, Johns Hopkins School of Medicine, Baltimore, Maryland, USA; d Department of Neuroscience, Johns Hopkins School of Medicine, Baltimore, Maryland, USA

## Abstract

In this study, we established induced pluripotent stem (iPS) cell lines from postmortem dura-derived fibroblasts of four control individuals with low polygenic risk score for psychiatric disorders including schizophrenia and bipolar disorder. The fibroblasts were reprogrammed into iPS cells using episomal vectors carrying *OCT3/4, SOX2, KLF4, L-Myc, LIN28* and shRNA-*p53*. All iPS cell lines showed the same genotype with parental postmortem brain tissues, expressed pluripotency markers, and exhibited the differentiation potency into three embryonic germ layers.

## Resource Table:

**Table:** 

Unique stem cell lines identifier	LIBDi002-A LIBDi006-A LIBDi007-A LIBDi009-A
Alternative names of stem cell lines	LIBD2c1 (LIBDi002-A) LIBD6c2 (LIBDi006-A) LIBD7c6 (LIBDi007-A) LIBD9c1 (LIBDi009-A)
Institution	Lieber Institute for Brain Development, Baltimore, USA
Contact information of distributor	Jennifer Erwin, Jennifer.Erwin@libd.org
Type of cell lines	iPSC
Origin	human
Cell Source	dural fibroblasts
Clonality	clonal
Method of reprogramming	episomal plasmids
Multiline rationale	control non-isogenic cell lines
Gene modification	NO
Type of modification	N/A
Associated disease	N/A
Gene/locus	N/A
Method of modification	N/A
Name of transgene or resistance	N/A
Inducible/constitutive system	N/A
Date archived/stock date	10-15-2019
Cell line repository/bank	https://hpscreg.eu/search?q=LIBD
Ethical approval	Western Institutional Review Board (WIRB), #WIRB 20111080

## 1. Resource Utility

The iPS cell lines generated from postmortem dura-derived fibroblasts of four non-psychiatric healthy individuals whose genotypic and transcriptomic data of multiple brain regions are available through BrainSEQ consortium can be differentiated into multiple types of brain cells by 2D and 3D methods and provide control lines to model neuropsychiatric disorders.

## 2. Resource Details

Recent progresses in induced pluripotent stem cell technologies have enabled the modelling of human brain development and investigation of molecular and cellular mechanisms underlying neurological/neuropsychiatric disorders *in vitro*. However, in most cases, it is difficult to access the clinical/pathological information obtained from the donor’s brains. BrainSEQ consortium (BrainSeq: A Human Brain Genomics Consortium. Neuron. 2015) is a project to characterize the genetic and epigenetic regulation of multiple facets of transcription in multiple brain regions across the human lifespan in samples of major neuropsychiatric disorders and controls using the Lieber Institute for brain development (LIBD)’s brain tissue repository (https://www.libd.org/brain-repository/). These comprehensive database and resources make it possible to model neuropsychiatric disorders with iPS cells derived from postmortem tissues and compare/validate the endophenotypes with the data from postmortem brains with the same donors.

**Figure 1.**
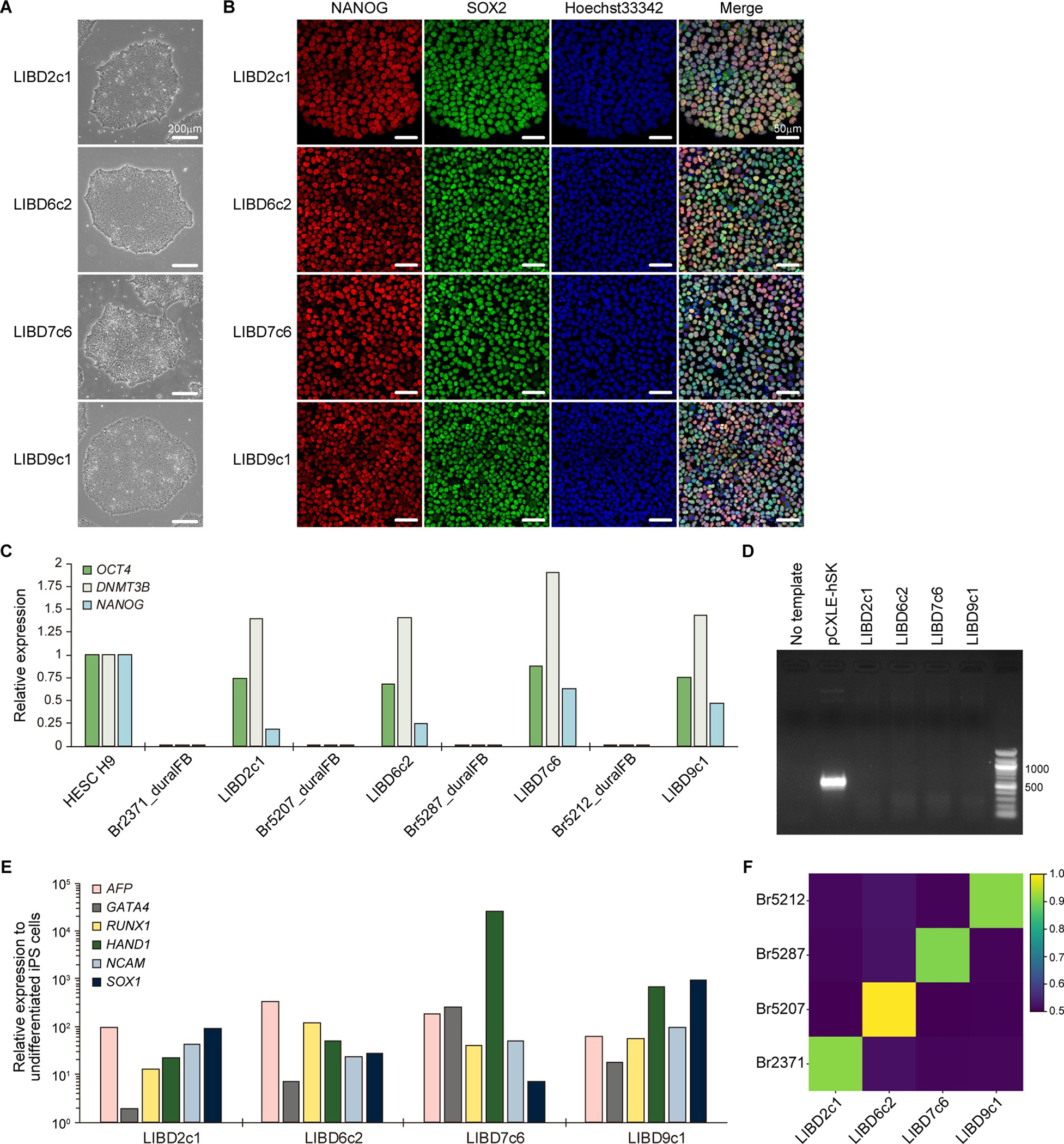
XXXX

In this study, we established iPS cells from postmortem dura-derived fibroblasts of non-psychiatric control individuals (Table 1) selected from the LIBD brain repository using episomal vectors carrying *OCT3/4, SOX2, KLF4, L-Myc, LIN28* and shRNA-*p53* and characterized them (Table 2). Each line exhibited typical human embryonic stem (ES) cell-like morphology (Fig. 1A). The expression of pluripotent stem cell markers, SOX2, OCT4, DNMT3B and NANOG, was confirmed by immunostaining (Fig. 1B) and/or RT-qPCR (Fig. 1C). We also confirmed the absence of genomic integration of episomal vectors utilized for reprogramming by genomic PCR (Fig. 1D). The pluripotency of established iPS cell lines was validated by *in vitro* spontaneous differentiation assay. RT-qPCR analysis revealed that the expression of three germ layer markers: α-fetoprotein (*AFP*) and *GATA4* (endoderm), *RUNX1* and *HAND1* (mesoderm) and *NCAM* and *SOX1* (ectoderm) was upregulated upon differentiation compared with undifferentiated iPS cells (Fig 1E). All lines were negative for mycoplasma (Supplementary Table 1).

Genomic integrity analysis was performed by copy-number analysis of genome-wide genotyping array at passage number 5 and each cell line was confirmed to contain 46,XY without any chromosomal abnormalities or copy number variations compared with hg19 reference genome (Supplementary Figure 1). As a note, this method will not detect balanced translocations. To confirm the identity of established iPS cell line, we compared DNA profile between established iPS cell lines and parental brain tissues (dbGaP: phs000979.v1.p1). Comparison of 1,478,103 single nucleotide polymorphisms (SNPs) of iPS cells with the original brains showed a similarity score (hamming distance) of at least >0.9 (Fig 1F) demonstrating that each iPS cell line is genetically identical to the donor’s postmortem brain tissue.

## 3. Materials and Methods

### 3.1. Case selection

Four Caucasian males without any psychiatric symptoms carrying low polygenic risk scores of psychiatric disorders (Wray et al., Genome Res. 2007; Schizophrenia Working Group of the Psychiatric Genomics Consortium. Nature. 2014) were selected from the LIBD brain repository.

### 3.2. Fibroblast culture

Human postmortem dural tissues were collected at autopsy and fibroblasts were generated in a previous study (Bliss et al., PLoS One. 2012). Dura-derived fibroblasts were grown in fibroblast medium consisting of DMEM + GlutaMAX (Gibco), 10% fetal bovine serum (Gibco), and 1 x Antibiotic-Antimycotic (Gibco) in the incubator (at 5% CO2 and 37°C). Cells were passaged by trypsinization with 0.25 x Trypsin-EDTA solution (Sigma) for 2 to 3 min at room temperature.

### 3.3. Generation of iPS cells

Dura-derived fibroblasts between passages 5 and 7 were reprogrammed with episomal vectors (Okita et al., Nat. Methods. 2011). Plasmids pCXLE-hOCT3/4-shp53-F (Addgene #27077), pCXLE-hSK (#27078), and pCXLE-hUL (#27080) were transfected into fibroblasts using 4D-Nucleofector system with P2 Primary Cell 4D-Nucleofector X Kit (Lonza; program DT-130). Three to five weeks after reprogramming, single colonies were picked and expanded on feeder cells (SNL76/7, mitomycin C-treated) in 20% KSR medium (Okita et al., Nat. Methods. 2011) or on Cultrex BME (Trevigen) in StemFlex medium (Gibco). iPS cells were passaged every 4–6 days with CTK solution (Okita et al., Nat. Methods. 2011) consisting of 0.25% trypsin, 0.1 mg/ml Collagenase Type IV, 1 mM CaCl2, and 20% KSR or Versene solution (Gibco). For every passaging, cells were split 1:4 to 1:6.

### 3.4. Immunocytochemistry

The feeder-free iPS cells were fixed with 4% PFA (Sigma) at room temperature for 10 min and permeabilized and blocked with 0.3% Triton X-100 (Sigma), 10% normal horse serum (Thermo Fisher) in DPBS (Gibco) at room temperature for 30 min. Cells were then stained with antibodies against SOX2 and NANOG (Table 3) diluted in 5% normal horse serum/0.01% Tween-20/D-PBS at 4°C overnight and incubated with fluorescent dye-conjugated secondary antibodies (Table 3) diluted in 0.01% Tween-20/D-PBS at room temperature for 2 h. Cell nuclei were labeled by Hoechst33342 (1:10,000, Invitrogen) for 10 min. Stained cells were images with a laser confocal microscopy (LSM700, Zeiss).

### 3.5. RT-qPCR

Total RNA was extracted with Direct-zol Mini prep kit (Zymo Research). For reverse transcription, we used SuperScript IV VILO Master Mix (ThermoFisher Scientific). The expression of pluripotent stem cell markers (Table 3) was analyzed via qPCR with QuantiTect SYBR Green PCR kit (Qiagen) on QuantStudio 3 (Applied Biosystems). Total RNA from a human embryonic stem cell line (HESC H9) (ScienCell) was used as a positive control and dural fibroblasts (FB) from each individual used as negative controls.

### 3.6. Genome integration analysis

DNA was extracted from iPS cells with DNeasy Blood and Tissue kit (Qiagen) according to the manufacturer’s instruction. PCR was performed with the OriP primers (Table 3), Platinum Taq DNA Polymerase (Invitrogen) and the following program: initial denaturation for 2 min at 94°C and 25 cycles of 94°C for 30 sec, 60°C for 30 sec, 72°C for 1 min on T100 Thermal Cycler (Bio-Rad).

### 3.7. in vitro spontaneous differentiation

iPS cells on feeder were harvested using CTK solution (Okita et al., Nat. Methods. 2011). The cell clumps were transferred to Ultra Low Attachment plates (Corning) in 20% KSR medium without bFGF. The medium was changed every other day. Twenty-day-old embryoid bodies (EBs) were harvested. The expression of three germ layer markers (Table 3) was analyzed via RT-qPCR.

### 3.8. Genome-wide genotyping array analysis

Genomic DNA was extracted from feeder-free iPS cells with DNeasy Blood and Tissue kit (Qiagen) according to the manufacturer’s instruction. Genotyping array analysis was performed by Macrogen with an Infinium Omni 2.5–8 kit (illumina).

### 3.9. Copy number variation analysis

Genome Studio (v2.0) was used to calculated log-2 represent probe intensity ratio (Log R) representing a ratio of observed intensity to reference intensity. Segmentation and plotting were generated using aspcf (kmin = 500) and plotGenome utilities from the Bioconductor package copynumber (Nilsen et al., R package version 1.26.0. 2013; Nilsen et al., BMC Genomics. 2012).

### 3.10. Genotype comparison between iPS cells and parental brain tissues

Genotyping with Illumina BeadChips was carried out using DNA extracted from cerebellar tissues and iPS cells. Low quality and rare variants were removed with PLINK. For parental tissues, genotypes were prephased and imputed using genome build hg19. Genotypes were matched between cells and brain tissues with hamming distance used to compare genomic similarity.

### 3.11. Mycoplasma detection

Mycoplasma test was carried out with MycoAlert mycoplasma detection kit (Lonza) according to the manufacturer’s instruction. The ratio of Reading B to Reading A is used to determine whether a cell culture is contaminated by mycoplasma with the following cutoff: ratio < 0.9 for negative and > 1.2 for positive.

## Supplementary Material

1

supplemental table 1

## Figures and Tables

**Table 1:** Summary of lines

iPSC line names	Abbreviation in figures	Gender	Age	Ethnicity	Genotype of locus	Disease

LIBDi002-A	LIBD2c1	Male	23	Caucasian	N/A	Control
LIBDi006-A	LIBD6c2	Male	61	Caucasian	N/A	Control
LIBDi007-A	LIBD7c6	Male	40	Caucasian	N/A	Control
LIBDi009-A	LIBD9c1	Male	51	Caucasian	N/A	Control

**Table 2:** Characterization and validation

Classification	Test	Result	Data

**Morphology**	Photography	Normal	Fig. 1 panel A
**Phenotype**	Qualitative analysis (Immunocytochemistry)	Positive for SOX2 and NANOG	Fig. 1 panel B
	Quantitative analysis (RT-qPCR)	Positive for *OCT4, DNMT3B* and *NANOG*	Fig. 1 panel C
**Genotype**	Genome-Wide Genotyping Array (Infinium Omni2.5)	No chromosomal abnormalities or copy number variations larger than 1MB	Supplementary figure 1; Submitted in archive with journal
**Identity**	Genome-Wide Genotyping Array (Infinium Omni2.5)	1,478,103 SNP sites tested, matched	Fig. 1 panel F
**Microbiology and virology**	Mycoplasma	Mycoplasma testing by luminescence: Negative	Supplementary Table 1
**Differentiation potential**	Embryoid body formation	Expression of *AFP* and *GATA4* (endoderm), *RUNX1* and *HAND1* (mesoderm), and *NCAM* and *SOX1* (ectoderm)	Fig. 1 panel E

**Table 3:** Reagents details

Antibodies used for immunocytochemistry/flow-citometry			
	Antibody	Dilution	Company Cat # and RRID

Pluripotency Markers	Rabbit anti-SOX2 (D6D9)	1:500	Cell Signaling Technology Cat# 3579, RRID: AB_2195767
	Goat anti-NANOG	1:200	R&D Systems Cat# AF1997, RRID: AB_355097
Secondary antibodies	Donkey anti-rabbit IgG Alexa 488 conjugated	1:250	Jackson ImmunoResearch Cat# 711-545-152, RRID: AB_ 2556546
	Donkey anti-goat IgG Cy3 conjugated	1:250	Jackson ImmunoResearch Cat# 705-165-147, RRID: AB_2307351

Primers		
	Target	Forward/Reverse primer (5′-3′)

Genomic integration of episomal vectors (PCR)	*OriP* (expected PCR product size: 590 bp)	TTCCACGAGGGTAGTGAACC/ TCGGGGGTGTTAGAGACAAC
Pluripotency Markers (qPCR)	*OCT4*	GGAAGGAATTGGGAACACAAAGG/ AACTTCACCTTCCCTCCAACCA
	*DNMT3B*	TGCTGCTCACAGGGCCCGATACTTC/ TCCTTTCGAGCTCAGTGCACCACAAAAC
	*NANOG*	CAGCCCCGATTCTTCCACCAGTCCC/ CGGAAGATTCCCAGTCGGGTTCACC
Differentiation Markers (qPCR)	*AFP* (endodermal)	AGCTTGGTGGTGGATGAAAC/ CCCTCTTCAGCAAAGCAGAC
	*GATA4* (endodermal)	CTAGACCGTGGGTTTTGCAT/ TGGGTTAAGTGCCCCTGTAG
	*RUNX1* (mesodermal)	CCCTAGGGGATGTTCCAGAT/ TGAAGCTTTTCCCTCTTCCA
	*HAND1* (mesodermal)	GAGAGCATTAACAGCGCATTCG/ CGCAGAGTCTTGATCTTGGAGAG
	*NCAM* (ectodermal)	ACATCACCTGCTACTTCCTGA/ CTTGGACTCATCTTTCGAGAAGG
	*SOX1* (ectodermal)	ATGCACCGCTACGACATGG/ CTCATGTAGCCCTGCGAGTTG
House-Keeping Genes (qPCR)	*GAPDH*	CATGAGAAGTATGACAACAGCCT/ AGTCCTTCCACGATACCAAAGT
