# Supplementary figures and images for: Generation of four postmortem dura-derived iPS cell lines from four control individuals with genotypic and brain-region-specific transcriptomic data available through the BrainSEQ consortium
Source: Stem Cell Res. Author manuscript; Available in PMC 2023 Sep 22. (PMC10515715; doi:10.1016/j.scr.2020.101806)

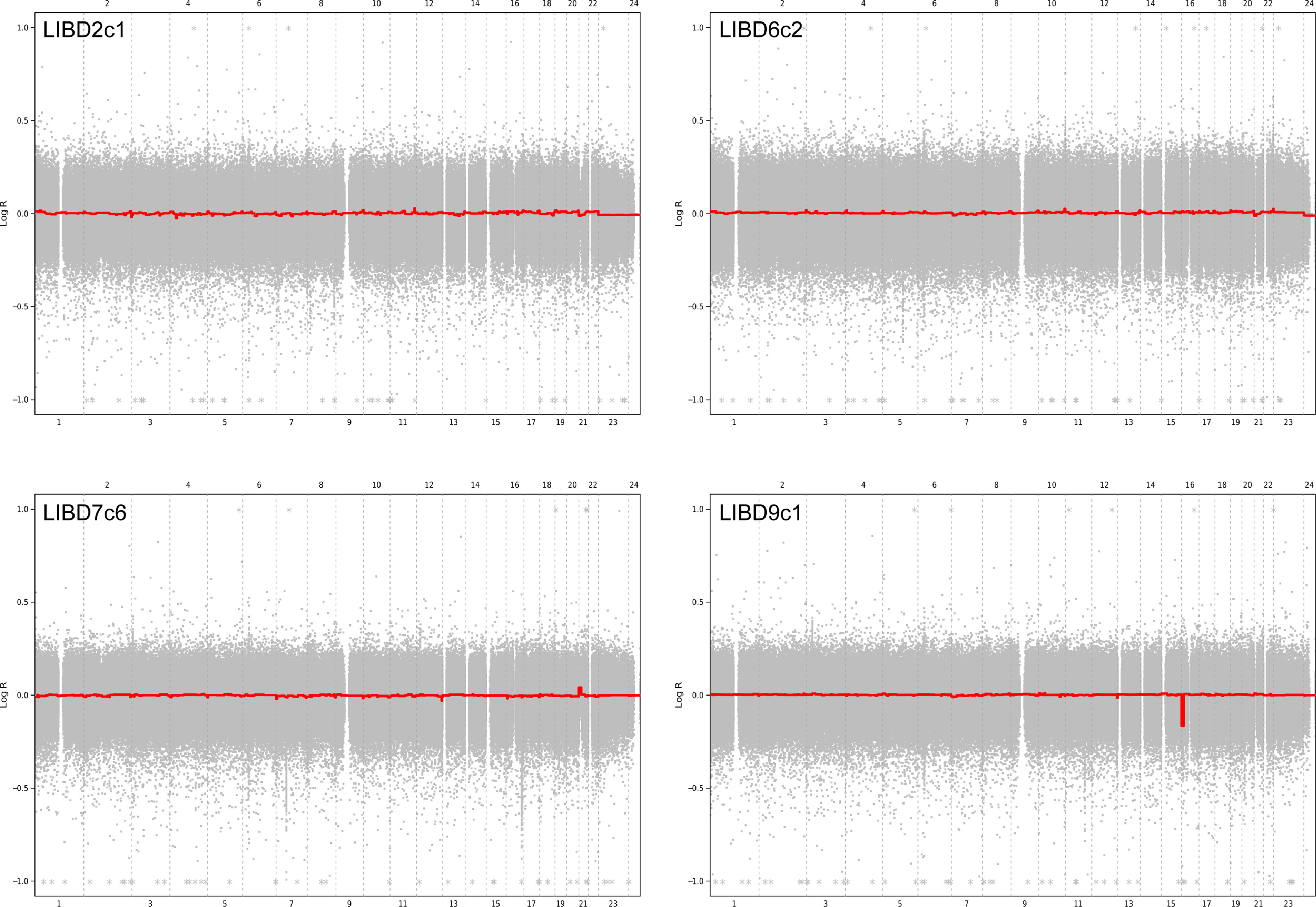

Supplement: 1 [file NIHMS1928902-supplement-1.jpg]
